# Oncogenic Potential of Bisphenol A and Common Environmental Contaminants in Human Mammary Epithelial Cells

**DOI:** 10.3390/ijms21103735

**Published:** 2020-05-25

**Authors:** Vidhya A Nair, Satu Valo, Päivi Peltomäki, Khuloud Bajbouj, Wael M. Abdel-Rahman

**Affiliations:** 1 Sharjah Institute for Medical Research, University of Sharjah, Sharjah P.O. Box 27272, UAE; vnair@sharjah.ac.ae (V.A.N.); kbajbouj@sharjah.ac.ae (K.B.); 2 Department of Medical and Clinical Genetics, University of Helsinki, FI-00014 Helsinki, Finland; satu.valo@blueprintgenetics.com (S.V.); paivi.peltomaki@helsinki.fi (P.P.); 3 Department of Medical Laboratory Sciences, College of Health Sciences, University of Sharjah, Sharjah P.O. Box 27272, UAE

**Keywords:** bisphenol A, breast cancer, hexabromocyclododecane, methoxychlor, nonylphenol, octylphenol

## Abstract

There is an ample epidemiological evidence to support the role of environmental contaminants such as bisphenol A (BPA) in breast cancer development but the molecular mechanisms of their action are still not fully understood. Therefore, we sought to analyze the effects of three common contaminants (BPA; 4-tert-octylphenol, OP; hexabromocyclododecane, HBCD) on mammary epithelial cell (HME1) and MCF7 breast cancer cell line. We also supplied some data on methoxychlor, MXC; 4-nonylphenol, NP; and 2-amino-1-methyl-6-phenylimidazo [4–b] pyridine, PhIP. We focused on testing the prolonged (two months) exposure to low nano-molar concentrations (0.0015–0.0048 nM) presumed to be oncogenic and found that they induced DNA damage (evidenced by upregulation of pH2A.X, pCHK1, pCHK2, p-P53) and disrupted the cell cycle. Some agents induced epigenetic (methylation) changes of tumor suppressor genes TIMP3, CHFR, ESR1, IGSF4, CDH13, and GSTP1. Obviously, the accumulation of these molecular alterations is an essential base for cancer development. Consistent with this, we observed that these agents increased cellular invasiveness through collagen. Cellular abilities to form colonies in soft agar were increased for MCF7. Toxic agents induced phosphorylation of protein kinase such as EGFR, CREB, STAT6, c-Jun, STAT3, HSP6, HSP27, AMPKα1, FAK, p53, GSK-3α/β, and P70S6 in HME1. Most of these proteins are involved in potential oncogenic pathways. Overall, these data clarify the molecular alterations that can be induced by some common environmental contaminants in mammary epithelial cells which could be a foundation to understand environmental carcinogenesis.

## 1. Introduction

Breast cancer incidence has been generally rising over the last 50 years with rapid increases observed particularly in developing countries [1,2,3]. Interestingly, developing countries show a higher incidence of breast cancer in urban compared to rural areas [4]. Furthermore, studies showed that women who migrate from low incidence to high incidence environment tend to develop breast cancer at higher rate than before supporting the hypothesis that breast cancer risk is strongly associated with environmental factors [5,6]. The Breast Cancer Fund report entitled “State of the Evidence: The Connection Between Breast Cancer and the Environment” released in October 2010 stresses upon the growing evidence linking breast cancer to a wide range of environmental exposures including bisphenol A (BPA) in food containers. Examples of other exposures include 4-nonylphenol (NP), 4-tert-octylphenol (OP), methoxychlor (MXC), and hexabromocyclododecane (HBCD); all of which could mimic estrogenic action and hence act as “endocrine disruptors” [7]. Additionally, environmental exposures include other contaminants of our food and drink such as the common food carcinogen 2-amino-1-methyl-6-phenylimidazo [4–b] pyridine (PhIP) which is a heterocyclic amine formed during the high-temperature cooking of meat and is known as a “cooked-meat mutagen” [8,9,10].

BPA is a synthetically produced chemical plasticizer that is commonly used in the production of polycarbonate plastics and epoxy resins which line various containers for foods, drinks, baby bottles, and water tanks. Human exposure to BPA occurs mainly through consumption of canned food and drinks because BPA migrates from the containers into the contents when heated or even under normal conditions of use. The endocrine disrupting action of BPA can be explained by its weak binding affinity for the estrogen receptors, ERα and ERβ [11,12]. BPA is also able to induce activation of Erk1/2 in human breast cancer cells through activation of G protein-coupled receptor 30 (GPR30) [13]. BPA can induce a complex set of signaling which cannot be explained by its binding to estrogen receptors only and it exerts many of these effects at low doses even lower than the current “safe” exposure limit for humans [14,15,16]. There is a strong evidence to implicate perinatal BPA exposure in mammary cancer development in various animal models and in vitro [7,15,17,18]. BPA is also able to induce resistance to doxorubicin, cisplatin, and vinblastine in vitro [19,20].

NP and OP are alkylphenols commonly used in production of laundry and dish detergents, emulsifiers, and solubilizers, as additives for fuels and lubricants, and are also used in making fragrances, antioxidants, and fire-retardant materials. Alkylphenols are xenoestrogens that can exert an endocrine disrupting effect with controversial carcinogenic effect which seems to be lineage specific [21,22,23]

MXC is a synthetic organochlorine used as an insecticide that was intended to be a replacement for DDT. It was banned in the European Union in 2002 and the US by 2003 based on its acute toxicity. It stimulates the growth of ovarian carcinoma cells in vitro by regulating cell cycle genes and abrogating apoptosis via an ER-dependent pathway [24]. MXC is detected at higher concentrations in breast cancer tissues compared to normal tissues [25] but a full understanding of detailed oncogenic mechanism is still lacking.

HBCD is one of the brominated flame retardants and is a major component of foam that is used as thermal insulation in the building industry, furniture, automobile interior textiles, car cushions, and packaging material. HBCD can induce DNA damage [26], exert endocrine disruption, and enhance growth of cancer cells of various lineages including prostate cancer [27] and ER-positive ovarian cancer [28]. Furthermore, HBCD induced chemo-resistance to cisplatin in a group of hepatocellular carcinoma cells (HepG2, MHCC97H, and MHCC97L) [29].

The role of cooked meat toxins such as PhIP in human breast cancer development is suggested by epidemiological studies and consequently the specific role of PhIP in breast cancer development is proved and attributed to estrogenic effect in some studies [30,31] but debated by others [32]. 

Thus, clear evidence of mammary cell oncogenicity together with the detailed oncogenic molecular pathways are still lacking for some toxic agents. Furthermore, many studies used already established breast cancer cell lines, which would be difficult to extrapolate on non-malignant (normal, immortalized, or benign) mammary epithelial cells. Therefore, this project aims to clarify the genotoxic/oncogenic effects of these toxic chemicals (with a focus on BPA, HBCD, and OP) in mammary epithelial cells and clarify the molecular pathways of these effects. This study is important to supply evidence-based approach to cancer prevention.

## 2. Results

### 2.1. Environmental Contaminants and Cell Viability

**Figure 1:**
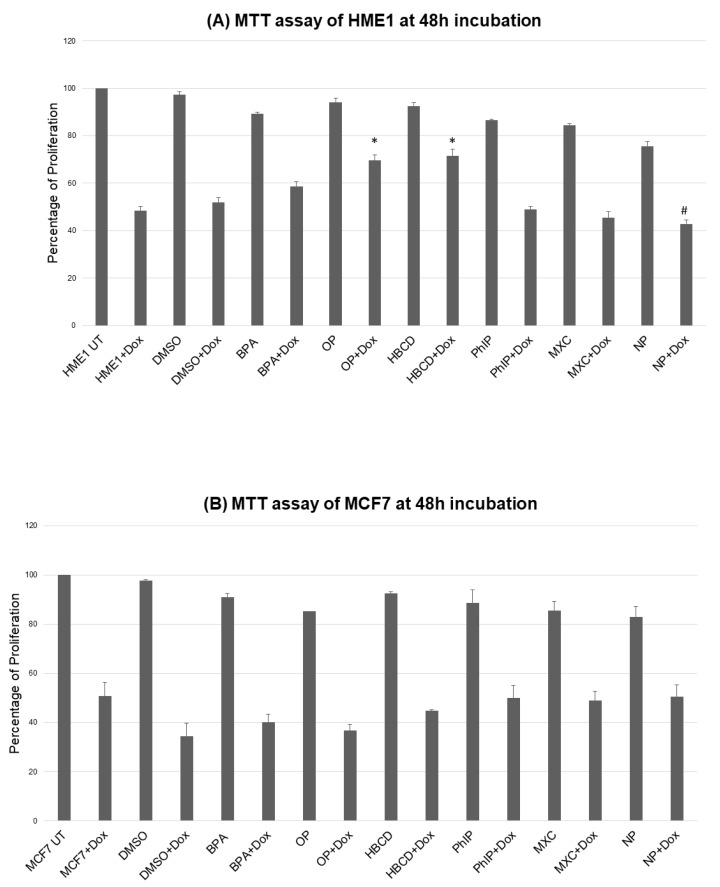
Representative graph showing altered viability of (**A**) HME1 and (**B**) MCF7 treated with nanomolar concentrations of various toxic agents (BPA, MXC, HBCD, NP, OP, PhIP at 0.0043, 0.0028, 0.0015, 0.0045, 0.0048, 0.0044 nM, respectively) alone or after overnight exposure to a therapeutic concentration of doxorubicin. Of note that, some toxic agents interfered with the killing effect of doxorubicin. Mean ± SEM, * *p* < 0.05, # indicates a tendency (*p* < 0.10), *n* = 3 (3 biological × 3 technical).

We first tested different concentrations of toxic agents for their effects on viability. In general, high concentrations (1–10 μM) reduced viability while exposure to low nanomolar concentrations of toxic agents (0.0015–0.0048 nM) exerted subtle effect on viability; and some counteracted the cytotoxic effect of doxorubicin as compared to control (vehicle-exposed) cells. Therefore, we rather focused on the low concentrations (BPA, MXC, HBCD, NP, OP, PhIP at 0.0043, 0.0028, 0.0015, 0.0045, 0.0048, 0.0044 nM, respectively) because of this potential to exert a pro-survival advantage after treatment with the cytotoxic agent doxorubicin (Figure 1). The focus of this work was to analyze the transformation potential on the mammary epithelium which is a long process that naturally might take years, so we opted for a prolonged exposure (2 months) after which we performed further functional analyses to dissect the potential long term transformation potentials of these toxic agents particularly on HME1 cells. 

### 2.2. DNA Damage by Environmental Contaminants 

**Figure 2:**
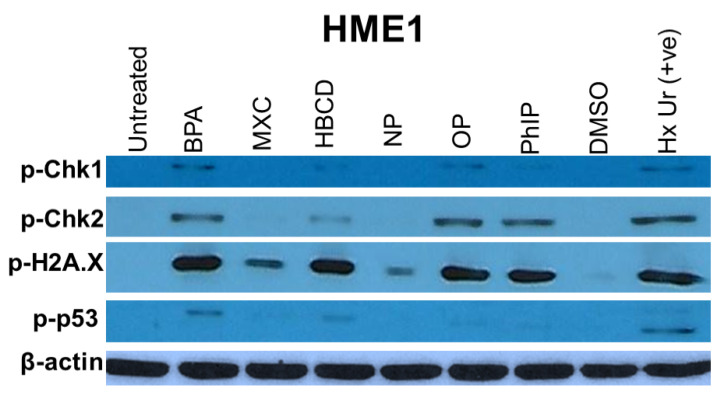
DNA damage response detected in the mammary epithelial cells HME1. Cells were treated with toxic agents (bisphenol A (BPA), methoxychlor (MXC), hexabromocyclododecane (HBCD), 4-nonylphenol (NP), 4-tert-octylphenol (OP), and 2-amino-1-methyl-6-phenylimidazo [4–b] pyridine (PhIP) at 0.0043, 0.0028, 0.0015, 0.0045, 0.0048, 0.0044 nM, respectively) for two months, then the expression of DNA damage response markers were tested by Western blot using antibodies against phospho-Chk1 (Ser345), phospho-Chk2 (Thr68), phospho-p53 (Thr18), and phosph-Histone H2A.X (Ser139). Markers were expressed to various levels in the cells exposed to these nanomolar concentrations of toxic agents for 2 months as compared to the untreated and DMSO control. Hydroxyurea (20 mM) was used as a positive control.

DNA damage detection has been optimized and performed using a selection of antibodies against phosphorylated DNA damage markers (p-Chk1, p-Chk2, p-histone H2A.X, p-p53) by Western blotting (Figure 2, Table 1). The experiment was performed on the two-month exposed cells to the low concentration. All 6 agents induced some DNA damage markers. Considering the total number of upregulated markers as a reflection of overall toxicity, the most DNA damaging agents were BPA and OP (8 markers each), followed by HBCD and PhIP (7 markers each), then NP (4 markers), and the least was MXC (3 markers).

### 2.3. Disruption of the Cell Cycle by Environmental Contaminants

Flowcytometry showed that exposure to environmental contaminants has multiple effects on the cell cycle. However, this effect was variable according to the cell lines, environmental contaminant and duration of exposures, these effects are given in details in Figures S1 and S2. Considering the results from cells exposed to environmental contaminants alone (without treatment with doxorubicin), we found that short exposure (24 h) to environmental contaminants induced mainly G2/M arrest in both MCF7 and HME1 with exception of OP in HME1 which increase in G1 peak. Other effects that were also noted after 24 h such as that BPA, MXC, HBCD, and OP caused increases in S phase in MCF7. After 48 h HME1was still showing the increase in G2/M but the MCF7 cell showed mainly increase in the G1 or S phases consistent with the expectation that cancer cells may have developed mechanisms to tolerate DNA damage. After 2 months exposure to these agents the MCF7 showed various effects according to each agent with BPA, MXC, HBCD, and NP causing G1 and S phase increases while OP caused G2/M and PhIP caused both S and G2/M phase increases; while HME1 showed an increase in S phase (all agents), G1 (MXC, PhIP), and G2/M (BPA, HBCD, NP, and OP).

It was also noted that exposures to environmental contaminants caused subtle reduction of the sub G1 peak (generally considered as a measure of apoptotic cells) that was induced by exposure to doxorubicin alone; this finding could support the chemo-resistance effect of these agents.

### 2.4. Methylation Changes in Tumor Suppressor Genes and LINE1

We performed these experiments on the cells exposed for two months using a commercial panel (MS-MLPA) which contains 24 well established tumor suppressor genes. Before any treatments, 15 of the 24 tumor suppressor gene loci were unmethylated in the MCF7 breast cancer cells. All loci except one (CDKN2B) were unmethylated in the untreated HME1 mammary epithelial cells. The vehicle control (dimethyl sulfoxide, DMSO) induced some methylation events and hence we reported the methylation in relation to this control. We observed that BPA induced hypermethylation of TIMP3, CHFR, ESR1, and IGSF4 while HBCD induced TIMP3 tumor suppressor gene hypermethylation in MCF7 breast cancer cell line. BPA additionally induced hypermethylation of CDH13 and GSTP1 in HME1 mammary epithelial cells. There were some instances of reduction of methylation of already methylated genes in MCF7 by BPA (CASP8, RASSF1, and GSTP1); therefore, we also tested LINE1 hypomethylation. LINE-1 methylation was slightly decreased in MCF7 treated with MXC, HBCD, NP, OP, and PhIP but Dm values remained above 0.70. Details of methylation changes by environmental contaminants are shown in Supplementary Table S1.

### 2.5. Environmental Contaminants and Collagen Invasion

**Figure 3:**
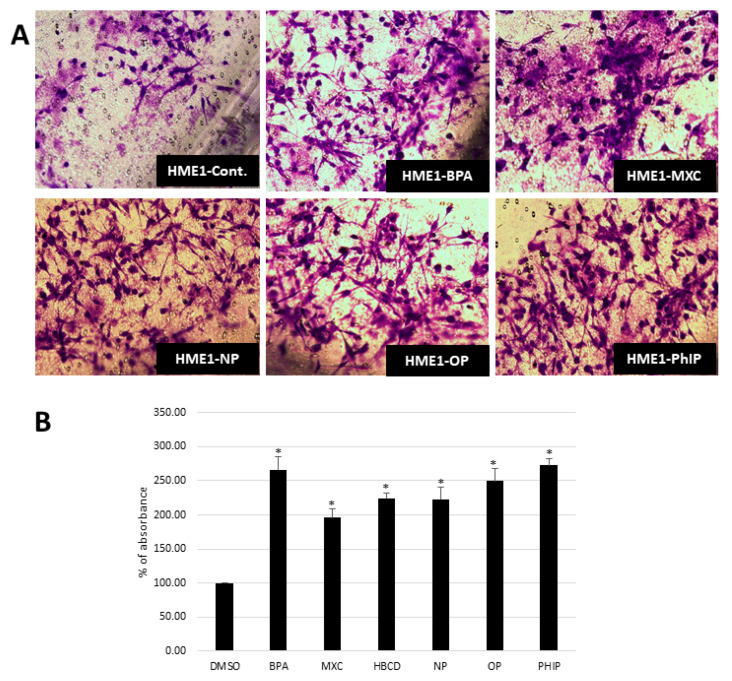
Collagen invasion assay of mammary epithelial cell (HME1) cell line exposed to various chemicals as compared to the base line invasion of control (vehicle-exposed) HME1 cell line. Cells were treated with nanomolar concentrations (BPA, MXC, HBCD, NP, OP, and PhIP at 0.0043, 0.0028, 0.0015, 0.0045, 0.0048, and 0.0044 nM, respectively) for two months then cells were serum starved and tested for their ability to invade through college-coated membrane using Boyden chambers. Invaded cells were fixed, stained, and visualized under the microscope and then the dye was eluted and measured using a plate reader. (**A**) Representative figures to show the differences in invasion potential as detected by microscopic examination. (**B**) quantitative evaluation of cell invasion as measured by dye elution and spectrophotometric reading at 560 nm. Mean ± SEM, * *p* < 0.05, *n* = 3.

In general, the results obtained by visual counting the number of invaded cells, matched the results obtained by spectrophotometry. Almost all agents increased cellular invasiveness to various degrees above the control (both untreated and vehicle; Figure 3).

### 2.6. Colony Formation in Soft Agar

Cell Transformation Detection Assay is an anchorage-independent growth assay in soft agar, which is considered the most stringent assay for detecting malignant transformation of cells. This experiment was performed on the 2-months-exposed cells. Some toxic agents showed increased colony formation above the control level in MCF7 cell lines Figure S3, however, none of the HME1 stocks formed colonies in soft agar under the test conditions. 

### 2.7. Proteomic Analysis (Human Phospho Kinase Array)

**Figure 4:**
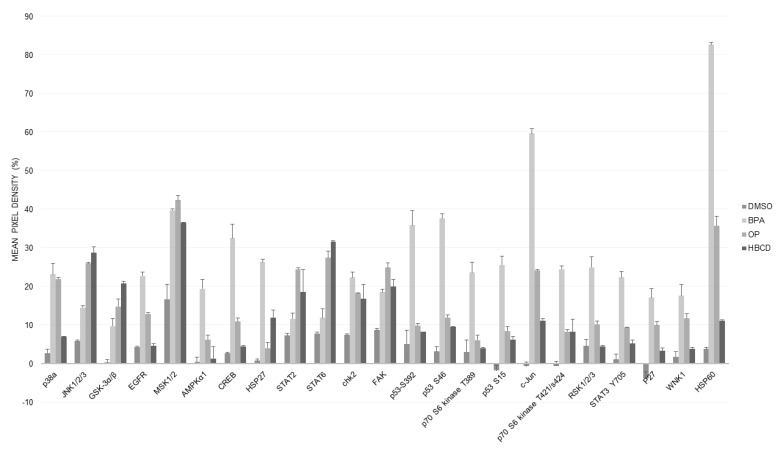
Phospho kinase array analysis after exposure of HME1 to low nanomolar concentrations of three agents (BPA, OP, and HBCD) for two months as compared to the vehicle (DMSO). Whole cell extracts were processed with “human phospho-kinase array” ready-made kit. The array analysis 43 kinase phosphorylation sites and 2 related total proteins with carefully selected specific capture antibodies. (Proteome Profiler; R&D Systems, Minneapolis, MN, USA). Data were analyzed using “image J” program and the Mean ± SEM was blotted as percentage (in relation to the positive control spot). Out of the 43 tested proteins, only the proteins which showed a change ratio of at least two times higher than that observed in the vehicle treatment (DMSO) are shown. *n* = 4 (2 biological × 2 technical).

In HME1 there was significant increase in 6 phosphoproteins: Epidermal growth factor receptor (EGFR), cyclic AMP response element binding protein (CREB), the signal transducer and activator of transcription 6 (STAT6), c-Jun, STAT3, and heat shock protein 60 (HSP60); and considerable increase in some others such as heat shock protein 27 (HSP27), 5′-AMP-activated protein kinase catalytic subunit alpha-1 (AMPKα1), Focal adhesion kinase (FAK), Glycogen synthase kinase 3 alpha/beta (GSK3α/β), Tumor protein p53 (p53), Ribosomal protein S6 kinase beta-1 (S6K1), also known as p70S6 kinase, ribosomal S6 kinase (RSK), Mitogen- and Stress-activated protein Kinases 1 and 2 (MSK1/2), p38 mitogen-activated protein kinases (p38α, MAPK14), and c-Jun N-terminal kinases (JNK1/2) (Figure 4). All of these were induced by either all three or at least two of the tested toxic agents (BPA, OP, HBCD). Interestingly, many of these activated phosphoproteins were not noticed in MCA7 which showed much less induction of phospho kinases as compared to HME1.

## 3. Discussion

In this work, we have generated data which might implicate exposure to low nanomolar concentrations of six common environmental contaminants in breast cancer development. 

Some of the tested agents interfered, to variable extent, with the cytotoxicity induced by doxorubicin, suggesting that they could predispose to chemoresistance. These data are consistent with, and adds emphasis on, the published work for BPA [19,20,33,34], the alkylphenols NP and OP [21], and PhIP [30]. Our data are also consistent with the effect of HBCD [35] in breast cancer. Interestingly, in some of the cited references above the described effect was induced by the smallest nanomolar doses. Collectively, these data suggest that low dose of these agents might play a role in breast cancer development through altered viability. These findings may also explain the increasing problem of chemoresistance in breast cancer. 

All toxic agents examined here showed potential to induce DNA damage as detected by over-expression of some established markers (pH2AX, pCHK1, pCHK2, p-p53). BPA and OP induced the highest number of DNA damage response markers suggestive of being the most extensive damage inducing agents. The DNA damage was also accompanied by cell cycle disruption marked mainly by arrest at G2/M phase which was bypassed after longer exposures in some instance. The survival of the cells with damaged DNA can predispose to malignant transformation by creating a state of “genomic instability” that accelerates acquisition of further oncogenic mutations [36].

Original mouse data described a pattern of epigenetic alteration dominated by hypomethylation of estrogen target genes, such as Hoxa10 due to low dose exposure to BPA in utero [37]. Global genomic hypomethylation was associated with BPA treatment also in other in vivo models such as zebrafish [38]. In the present work, we used a selected panel of 24 tumor suppressor genes. Our findings showed that some agents induced tumor suppressor gene hypermethylation in this panel. 

BPA induced hypermethylation of TIMP3, CHFR, ESR1, and IGSF4 while HBCD induced TIMP3 tumor suppressor gene hypermethylation in MCF7 breast cancer cell line. BPA also induced hypermethylation of CDH13, and GSTP1 in HME1 mammary epithelial cells. In addition, a few loci that were heavily methylated in untreated MCF7 cells decreased in methylation after treatments. Our pattern of hypermethylation and hypomethylation of selected genes resembles that reported by Fernandez et al. (2012) based on MeDIP-on-chip experiments on BPA-exposed MCF-10F breast epithelial cells [39]. Several of those tumor suppressor gene loci that we found hypermethylated after exposure to toxic agents showed frequent hypermethylation in breast cancer cohorts studied previously [40]. Our LINE-1 experiments did not reveal any significant hypomethylation when exposed to toxic chemicals (some mild hypomethylation could be seen).

Interestingly, the accumulated DNA damage and epigenetic alterations, both of which represent an essential base for cellular transformation and cancer development, were accompanied by features of transformed phenotype such as increased cellular invasiveness consistent with some published reports [15,30,31,41,42]. Recent literature suggested that BPA can disrupt mammary epithelial cells organization into acinar structures [43], We also observed some increased cellular abilities to form colonies in soft agar (anchorage independent growth) in MCF7 while this effect was not noted in HME1 at the test conditions. Cellular transformation might take prolonged periods and/or increasing doses, or may be, exposure to combination of toxic agents or toxic agents and other condition such as obesity or chronic inflammation. A recent study showed that combined exposures to two of the used agents here (BPA and NP) produced larger effects than the effect of each component applied separately, which could be the case in human life [44]. Even though our in vitro results are unable to explain how these dynamics might work in vivo, it would be difficult to take the lack of colonies formation in HME1 as an evidence against the oncogenicity of these agents in the presence of the accumulation of genetic alterations and increased cellular invasiveness of these cells. Furthermore, the literature data supply multiple evidence about the oncogenicity of BPA and many of these agents [15,30,42,45].

The phosphokinase array data uncovered many phosphoproteins and cellular pathways activated during cellular response to these toxic agents particularly in the mammary epithelial cells (HME1). We can generally classify these phosphoproteins into three related groups which form intricate network linking stress response to carcinogenesis. First and most important, many of the identified proteins play a well-established or putative oncogenic role in different stages of mammary carcinogenesis. Examples of these include EGFR, MSK1/2, CREB, FAK, GSK3α/β, S6K1/RSK, STAT2, STAT3, and STAT6. MSK1/2 are required for EGF-induced, histone H3-Ser10 phosphorylation and promoter-associated CREB phosphorylation and hence, early c-fos transcription in response to EGF [46]. The detection of these three phosphorylated proteins (EGFR, MSK1/2, CREB) strongly supports a functional consequences marked by EGF pathway transcriptional activity in response to toxic agents which could be an important early step in breast cancer development [47,48]. FAK is a cytoplasmic tyrosine kinase that plays critical role in integrin-mediated signal transductions. The activated FAK forms a complex with Src kinases family, which initiates multiple downstream signaling. FAK plays a critical role in cell migration and angiogenesis during cancer progression. EGF/IGF-I induced a co-localization of IGF-IR and FAK, which was evident mostly at the cell membranes of MCF-7 breast cancer cell and caused cell adhesion onto fibronectin, a marker of aggressive breast cancer [49]. The inhibitory phosphorylation of GSK3β might enhance cell proliferation and migration via β-catenin/cyclin D1 signaling pathway in breast cancer [50,51]. Both of p70S6 kinase and STAT3 could exert oncogenic potential in breast cancer through a phosphatidylinositide 3-kinases (PI3K)/Protein kinase B (Akt/PKB)/p70S6 kinase signaling pathway [52,53] while RSK is a family of Ser/Thr protein kinases (RSK1-4) that function downstream of the Ras/mitogen-activated protein kinase (MAPK) signaling to trigger cell survival, growth, and proliferation. Deregulated RSK activity has been associated with multiple cancer types including breast cancer [54]. 

The second group of induced proteins includes AMPKα1, p38α/MAPK14, JNK1/2, c-Jun, MSK1/2, CREB, HSP27, and HSP60 all of which could have a role in cellular stress and/or heat shock response. However, more recently, many of these proteins turned out to play an important role in carcinogenesis through an intricate network. The kinases MSK1 and MSK2 are linked to the epidermal growth factor-pathway as discussed above while the p38/MSK1/CREB transcription was shown to have oncogenic potential in some types of cancer [55]. In contrast to the earlier idea that HSPs are induced as a consequence of oncogene activation in cancer, the available literature data suggest that HSP60 is an early biomarker during malignant transformation in many cancers such as those of the breast and colon [56,57]. Researchers showed that bacterial and human HSP60 upregulate EGF, ERK, JNK, and p38/epithelial MAP kinase pathway [58,59]. Mutant EGFR showed the ability to induce HSPs such as HSP60 in lung carcinoma cells [60] suggesting a bidirectional positive feedback loop between HSP60 and EGFR. HSP60 was also reported to interact with and activate β-catenin [61], and can act as a ligand to Toll like receptor 4 (TLR4) that can activate the NF-κB pathway which is implicated in tumor development through creating a state chronic inflammation that favor tumor growth [62]. Similarly, an accumulating body of evidence supports a role of HSP27 in carcinogenesis [63].

The third group includes some DNA damage response and DNA repair proteins (p53, Chk2, and p27) which is consistent with and confirms our Western blot data described above. 

Some of the pathways mentioned above such as STAT3, EGFR/ERK, HSPs, and DNA repair were reported before in the context of exposure to environmental toxins particularly BPA [42], while the rest of the kinases identified in the current work may be reported for the first time here. Additional oncogenic pathways and mechanisms were reported in the literature. BPA was shown to reduce retinoblastoma-associated protein and increase the Rac1 and CDC24 in normal mouse mammary cultures [64]. BPA was also shown to promote angiogenesis through up regulation of X-linked inhibitor of apoptosis via G protein-coupled estrogen receptor pathway [65].

Overall, the available data supply an important evidence to implicate very low doses of six common environmental contaminants in inducing genotoxic/oncogenic alterations in mammary epithelial cells. We elucidated significant pathways upregulated by environmental contaminants which help understanding the mechanisms of action of these toxic agents. These data may explain the remarkable increase in breast cancer incidence and chemoresistance worldwide.

## 4. Materials and Methods 

### 4.1. Cell Lines

MCF-7 cell line was isolated from malignant metastatic pleural effusion of a woman having breast adenocarcinoma. It retains several ideal characteristics particular to the mammary epithelium e.g., positivity for estrogen receptors (ER) and cytokeratin while non-reactive to desmin and vimentin (mesenchymal markers). When grown in vitro, MCF7 can form domes and the epithelial like cells that grow in monolayers with doubling time of 38 h. MCF7 had a wild type p53 gene sequence (American Type Culture Collection, ATCC, Manassas, VA, USA). For this experiment, it was cultured in phenol red free DMEM supplemented with, 1mM Sodium Pyruvate, 10% char coal stripped-fetal bovine serum and 1x Penicillin and Streptomycin (Sigma-Aldrich, ST. Louis, MO, USA). hTERT-HME1 (HME1) was from Horizon Discovery (Cambridge, United Kingdom). It was derived from human normal mammary epithelial cells immortalized by stable transfection of hTERT telomerase. It was initially cultured as per the supplier conditions then maintained for the purpose of this experiment in phenol red free DMEM/F12-ham supplemented with 10% char coal stripped-fetal bovine serum and 1x Penicillin and Streptomycin (Sigma-Aldrich). All cells were maintained at 37 °C in a humidified 5% CO_2_ incubator and were split before their confluency reached 90% according to supplier instructions.

### 4.2. Toxic Chemicals and Viability Assays 

Environmental chemicals tested here were selected on the basis that they have a potential endocrine disrupting effect and/or that they were previously implicated in cancer development. These included BPA, NP, OP, MXC, HBCD, and PhIP. Chemicals were purchased from Sigma-Aldrich Corp. (BPA, NP, OP, and HBCD), Supelco, Bellefonte, PA, USA (MXC); or MP Biomedicals LLC, Solon, OH, USA (PhIP). Chemicals were dissolved in 100% dimethyl sulfoxide or as recommended and stored as stock solutions at 4 °C. Initially we performed a pilot study to find out the effect of increasing doses of these agents on viability using the standard MTT or Sulforhodamine B (SRB) method as described earlier [66]. For the two-month exposures, cells were seeded in T25 tissue culture flasks without chemicals or DMSO over night to settle and attach then chemicals or DMSO were added next morning. The DMSO concentration did not exceed 1%. Media with fresh chemicals were replaced every 3 days. Cells were split before they reached 90% confluency. All compounds tested concurrently. Stocks of exposed cells were frozen down in liquid nitrogen after two months. Toxic agents-exposed cells were also tested for viability after overnight exposure to doxorubicin. The IC50 concentration of Doxorubicin was determined as before [66]. Cells were seeded in triplicates in each plate. 

### 4.3. Detection of DNA Damage Response by Western Blot Analysis

Cell lysates were made from cultured cells washed twice with ice cold 1X PBS then the pellets were lysed using pre-chilled Triton lysis buffer (25 mM Tris HCl, pH 7.4, 150 mM NaCl, 1 mM EDTA, 1% Triton) to which (50 mM NaF, 1 mM Na3VO4, 1 mM PMSF and 2% Protease inhibitor) were added freshly, incubated on ice for 5 min and centrifuged at speed of 14,000 rpm for 10 min at 4 °C to remove the cell debris. Protein concentration was quantified using the BCA protein assay (Thermo Fisher Scientific, Waltham, MA, USA). Approximately 30 µg of total protein were subjected to SDS-PAGE (7.5% resolving gel, 4% stacking gel). The gel, with the separated proteins, was transferred to a PVDF membrane (Bio-Rad Laboratories, Hercules, CA, USA) using a Trans-Blot^®^ TurboTM Blotting system (Bio-Rad) according to the standard transfer protocol. The membrane was blocked for 1 h at room temperature using 5% non-fat dry milk (Sigma-Aldrich) in 1x TBST (TBS + 1% tween), followed by incubation with the primary antibody overnight at 4 °C on a shaker. Phospho-antibodies were against phospho-Chk1 (Ser345) clone 133D3, phospho-Chk2 (Thr68) clone C13C1, phospho-p53 (Thr18) and phosph- Histone H2A.X (Ser139) clone 20E3 together with their matching antibodies against total proteins, Beta-actin (clone 13E5). All antibodies were from Cell Signaling Technology (Danvers, MA, USA) except phospho-p53 was from Santa Cruz Biotechnology (TX, USA). Primary antibodies were used at 1:1000 or at recommended dilutions by suppliers. Next, the membrane was washed with 1x TBST 3 times for 10 min each. Then, it was incubated with horseradish peroxidase labeled secondary antibody for 1 h at room temperature (anti-rabbit IgG HRP-linked antibody at dilution of 1:2000, Cell Signaling). After washing with 1x TBST for 10 min 3 times, the HRP labeled membrane was incubated with substrate (Pierce™ ECL Western Blotting Substrate, Waltham, MA, USA) for 1 min, exposed to film (Kodak Cl-XPosure TM Film; catalog No: 34090; Company: Thermo Scientific), developed and fixed. Pictures were then evaluated and scanned.

### 4.4. Analysis of DNA Content and Cell Cycle Progression

Cells were seeded at a density of 1 × 10^6^ cells/mL. After indicated timings, cells are harvested, washed twice with PBS, and then re-suspended in 0.5 mL ice-cold PBS and later fixed with 4  mL ice cold 70% ethanol for 48 h. Cells were then pelleted, washed twice with ice-cold PBS, re-suspended and incubated at room temperature for 30 min in 0.2 mL staining buffer supplemented with 50 μg RNase and propidium iodide (Sigma-Aldrich) at 50 μg/mL final concentration in the dark. Distribution of cell cycle phases with different DNA contents Cells was analyzed by flowcytometry (BD FACS Aria™ III, Becton-Dickinson, Franklin Lakes, NJ, USA). Analysis of cell cycle distribution and percentage of cells in G1, S, and G2/M phases of the cell cycle were determined using the cell cycle platform of the FlowJo software v10.6.2 with the Watson pragmatic model (Tree Star, Ashland OR, USA).

### 4.5. Methylation Specific Multiplex-Ligation Dependent Probe Amplification (MS-MLPA)

We used SALSA MS-MLPA Tumor suppressor Kit ME001-C1 (MRC, Amsterdam, Holland) to study promoter methylation of 24 tumor suppressor genes (TSGs) in tumor and normal samples according to manufacturer’s instructions (http://www.mrc-holland.com). This kit contains 26 probes for 24 TSGs, which were described in detail in our previous publication [67]. For each reaction, 200–800 ng of DNA was used. Each MS-MLPA reaction generates two aliquots, one undigested and one digested and these can be used for copy number and methylation detection respectively. MS-MLPA utilizes methylation sensitive enzyme *HhaI* and probes have a recognition sequence against this enzyme. Only templates with methylated HhaI sites, resistant to HhaI cleavage, generate a signal after probe ligation. Amplification products were separated by capillary electrophoresis and analyzed with ABI Prism GeneMapper 4.0 (Applied Biosystems, Waltham, MA, USA). In every assay, normal DNA derived from healthy individuals’ lymphocytes was included as a control for the lack of methylation and tumor cell line (RKO) was included as a reference for frequent methylation. Methylation dosage ratios were calculated as described in our previous study [67], in which a value of 0.15 (corresponding to 15% of methylated DNA) was used as cut-off level for methylation. This analysis was performed on the two-month exposed cells to the low concentration of all agents. 

For LINE-1 hypomethylation analysis (MS-MLPA) we followed the protocol described in our previous publication [68]. The custom designed probes target three different areas inside the LINE-1 promoter sequence (L1-1m, L1-2m, L1-3m). These sites were specified in the same reference. Whole genome amplified (WGA) DNA was used as a technical control for low LINE-1 methylation. WGA controls showed decreased LINE-1 methylation below the threshold of 0.70.

### 4.6. Invasion Assay

For the invasion assay, we used QCMTM High Sensitivity Non-cross-linked Collagen Invasion Assay kits (Merck group, Darmstadt, Germany) and followed the supplied manufacturer protocol. Briefly, the assay was performed using a modified chamber with filter inserts (pore size 8 μm) coated with matrigel in 24-well dishes. Approximately 0.5 million cells were prepared in the corresponding serum-free media. Two hundred and fifty microliters of the cell suspension were added into the inserts (top chamber) and 500 μL of 15% FBS-containing media were added to the bottom chamber. After 48-h incubation, cells remaining in the top chamber were removed and 400 μL of cell stain were applied to the invasion chamber insert for 15 min. After several washes with water, the inserts were dried, viewed under the microscope, photographed, and the numbers of invaded cells were counted/5 fields each 200× magnification. Inserts were then transferred into 200 μL of extraction buffer and allowed to incubate for 15 min at room temperature. The dye mixture was then assessed by a plate reader at a wavelength of 630 nm. 

### 4.7. Colony Formation in Soft Agar

Colony formation was analyzed using commercial kits (Cell Transformation Detection Assay; Merck group, Darmstadt, Germany). Cells pre-treated with carcinogens or control cells were cultured with appropriate controls in soft agar medium for 21–28 d. HeLa was used as a reference/positive control while the DMSO exposed cells were used as negative control. The, formed colonies were analyzed and counted morphologically using cell stain solution. This analysis was performed on the two-month exposed cells to the low concentration of all agents. 

### 4.8. Proteome Profile/Human Phospho-Kinase Array 

For molecular pathway analysis we used a “human phospho-kinase array” ready-made kit which analyzes alterations of kinase signaling in response to exposure to selected three agents (BPA, OP, HBCD) which showed the most significant response at most of the previous tests. The array analyses 43 kinase phosphorylation sites and 2 related total proteins with carefully selected specific capture antibodies. The experimental technique and analyses were according to manufacturer’s booklet which also contains a list of the targets and phosphorylation sites (Proteome Profiler; R&D Systems, Minneapolis, MN, USA). This analysis was performed on the two-month exposed HME1 cells to low concentration of BPA, OP, HBCD and vehicle (DMSO). selected results were confirmed by Western blot.

### 4.9. Data Analysis:

We used analysis of variance (one-way ANOVA) for mean comparisons. When the ANOVA test showed statistical differences, the Tukey post hoc test was used to discriminate between groups. *t*-Test was used to calculate significance of 2 independent means. *p* value of < 0.05 was considered to determine statistical significance and 0.05 < *p* < 0.10 indicated a tendency.

## Figures and Tables

**Table 1:** Induction of DNA damage markers by environmental contaminants.

	p-Chk1		p-Chk2		p-Histone H2A.X		p-p53		Total Positive Markers
	HME1	MCF7	HME1	MCF7	HME1	MCF7	HME1	MCF7	
**BPA**	++	++	++	++	++	++	++	++	8
**MXC**	-	-	-	+	+	-	-	+	3
**HBCD**	+	+	+	++	++	-	+	+	7
**NP**	-	++	-	++	+	-	-	++	4
**OP**	+	++	++	++	++	+	+	+	8
**PhIP**	+	++	++	++	++	-	+	+	7
**Total**	9		10		8		10		

Footnote: The gray highlight indicates the positive findings included in the “total”. +, weak expression; ++ strong expression, -, no expression.

## Supplementary Materials

Supplementary materials can be found at https://www.mdpi.com/1422-0067/21/10/3735/s1.
