# Supplementary material for: Oncogenic Potential of Bisphenol A and Common Environmental Contaminants in Human Mammary Epithelial Cells
Source: Int J Mol Sci. 2020 May 25;21(10):3735. doi: 10.3390/ijms21103735 (PMC7279350; doi:10.3390/ijms21103735)
Supplement: Supplementary file 1 [file ijms-21-03735-s001.zip › Supp Fig S#1 - HME1 cell cycle .pdf]

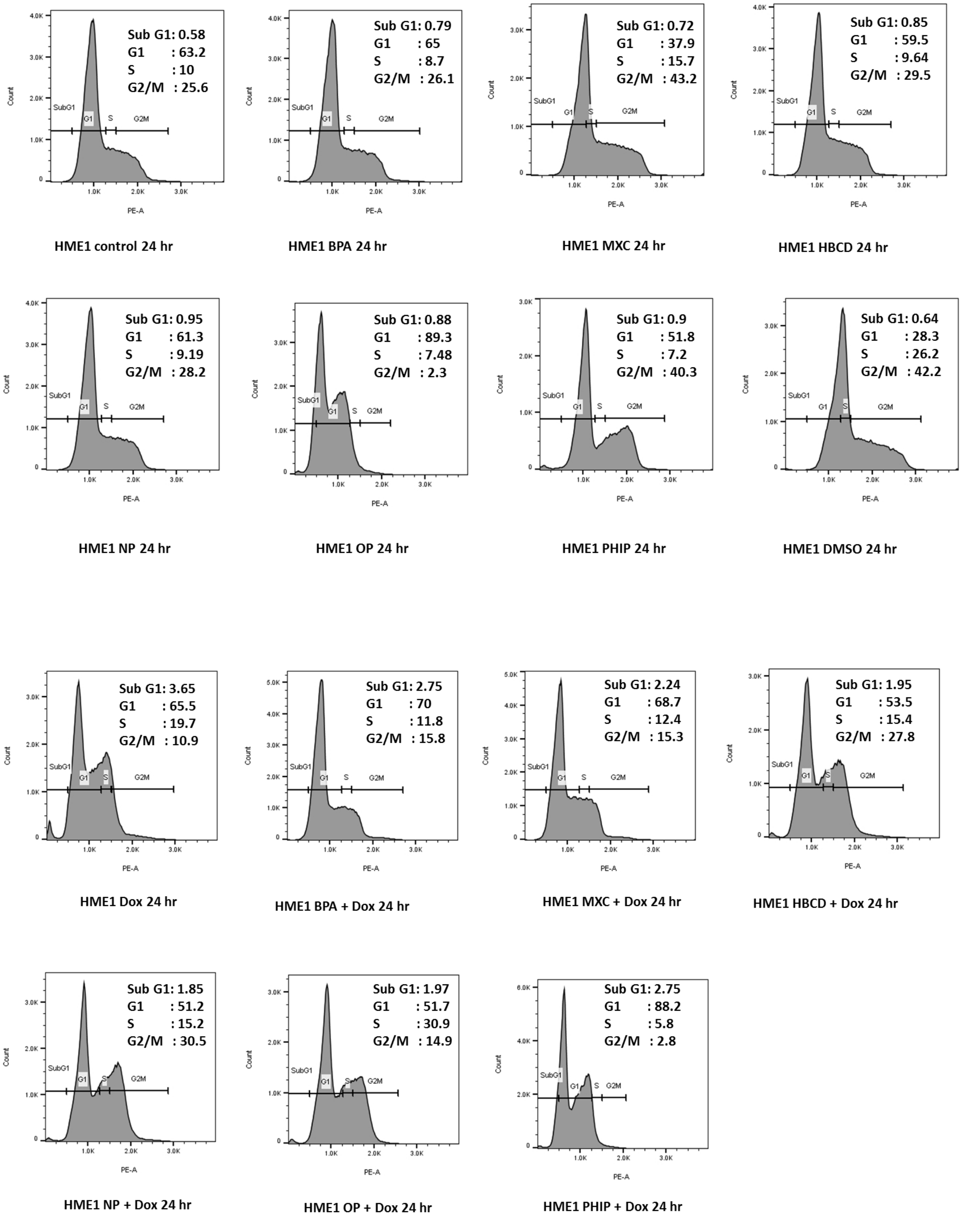

Figure 1 : Cell cycle analysis of HME1 at 24 hr incubation

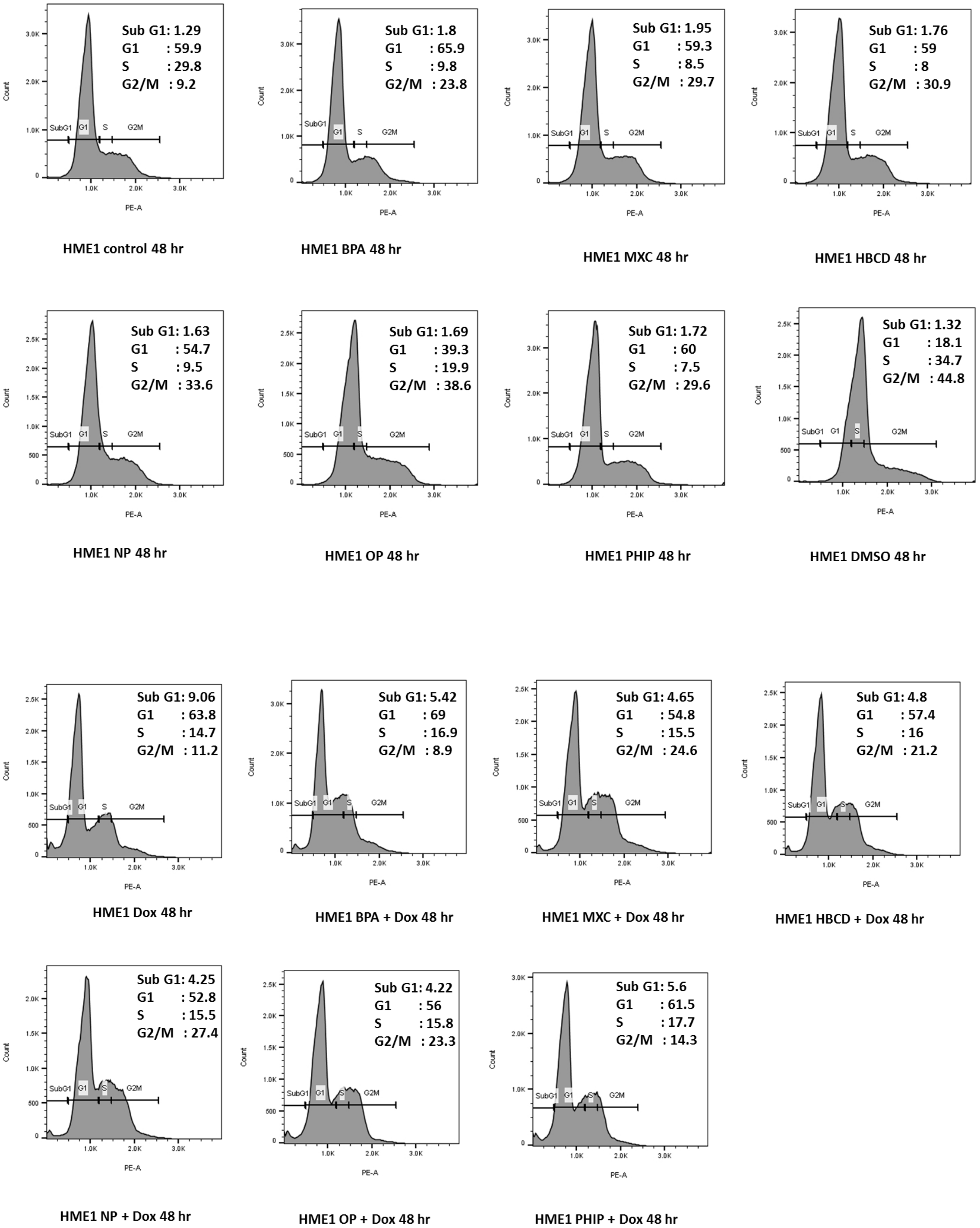

Figure 2 : Cell cycle analysis of HME1 at 48 hr incubation

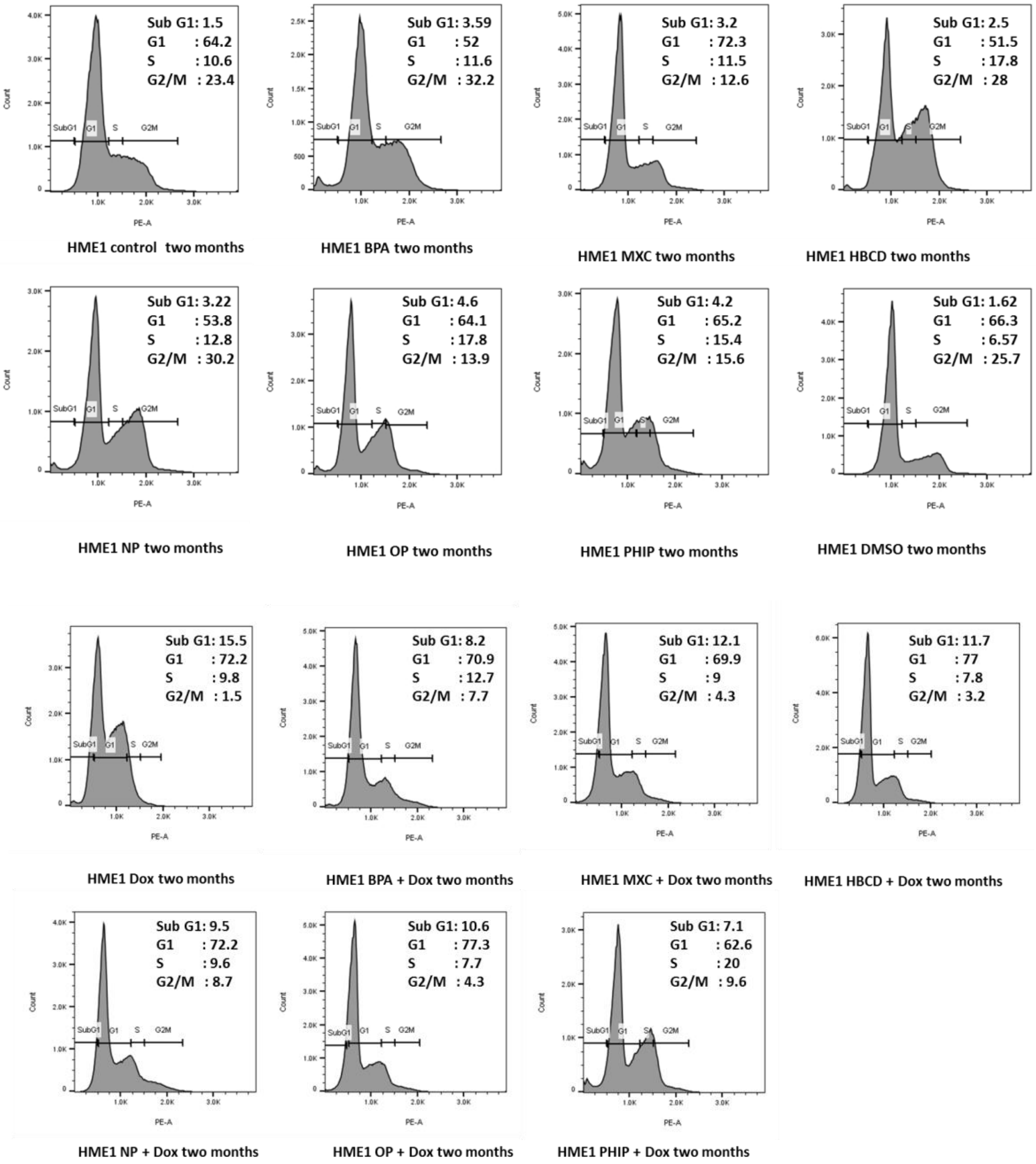

Figure 3 : Cell cycle analysis of HME1 at two months incubation
