# Supplementary figures and images for: Oncogenic Potential of Bisphenol A and Common Environmental Contaminants in Human Mammary Epithelial Cells
Source: Int J Mol Sci. 2020 May 25;21(10):3735. doi: 10.3390/ijms21103735 (PMC7279350; doi:10.3390/ijms21103735)

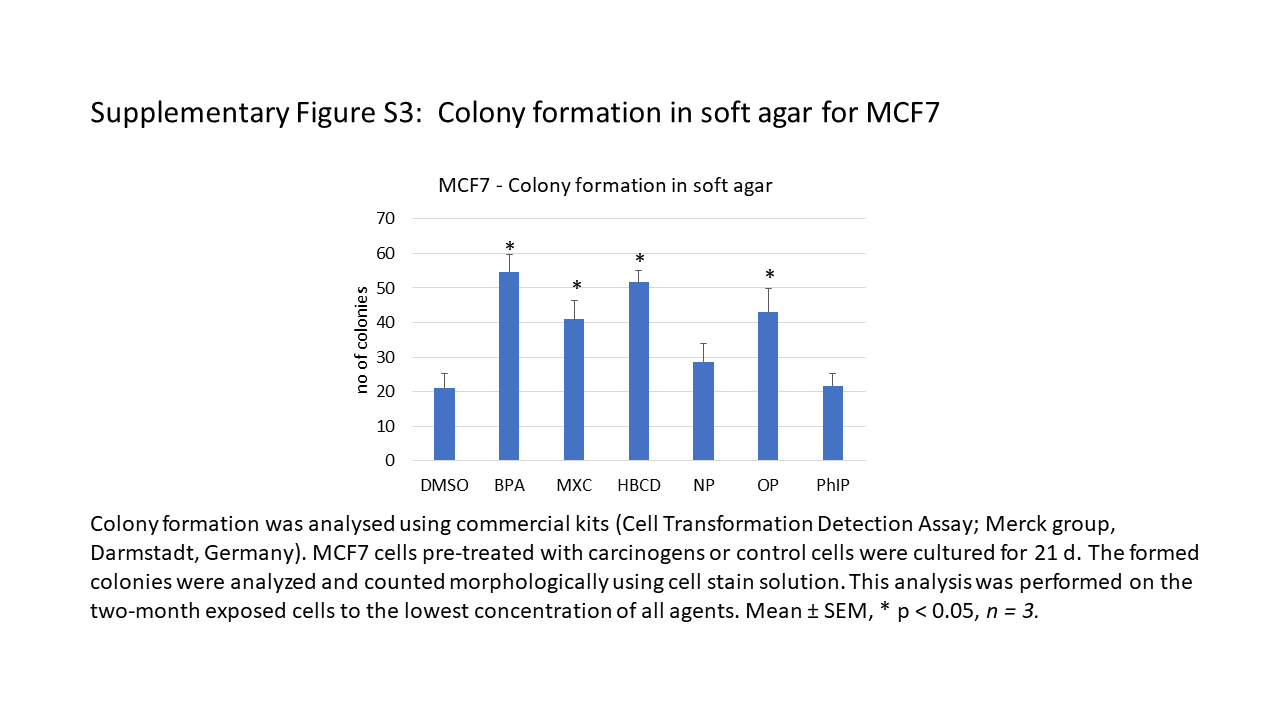

Supplement: Supplementary file 1 [file ijms-21-03735-s001.zip › Supplementary Figure S3.tif]
